# The Crusade against Venereal Disease

**Published:** 1917-06-30

**Authors:** 


### THE CRUSADE AGAINST VENEREAL DISEASE.

### Addresses by Surg.-General Sir Alfred Keogh and Mrs. Creighton.

GIVEN AT THE SECOND ANNUAL MEETING OF THE NATIONAL COUNCIL FOR COMBATING VENEREAL DISEASE.

### Surg.-General Sir Alfred Keogh, K.C.B., expressed his warm appreciation of what the National Council had done for the military Forces. The group of diseases under consideration had often been spoken of as if they were peculiar to the Army, but against that he protested. The idea probably arose from the fact that in the Army alone it had hitherto been possible to study the diseases statistically. It was desirable that the public should know the progress which had been made in the Army in this matter during the last few years. In the years 1905 and 1913 the contrast was very marked. In 1905, in the United Kingdom alone, the admission rate into the Army hospitals was 90 per 1,000; in 1913 the figure had fallen to 50 per 1,000. In India the admission rate was 154 per 1,000 in 1905; and in 1913 it had fallen to 52 per 1,000. For many years the admission rate for these diseases at Aldershot was the lowest of any military station in the United Kingdom—29 per 1,000, as against a fairly steady rate for London of 95 per 1,000. The campaign in the Army against these diseases began with Lord Roberts, and it was continued by Lord Kitchener. In India the good advance could never have been made but for the strong support given by the authorities there; and the achievement was mainly due to education. The men were told, in a plain way, as much as they ought to know about the pathology and the ravages of these diseases. And at the same time better facilities for recreation were provided. Nothing, he believed, approached education among the agencies for combating venereal diseases. He had lived all his life in the Army; he had treated many thousands of cases, and had conversed with very many men on the subject, and found an astonishing number of the victims were quite innocent, and very many fell into the danger through lack of recreation to occupy the time not spent in military duties. He had very gladly acquiesced in the suggestion made to him by Sir Thomas Barlow on behalf of the National Council to deliver lectures to the men of the New Army, and Lord Sydenham had already said that 1¼ million men had listened to them. Though it was a pleasure to mention the reduction of the incidence-rate of these diseases in the Army, it was no light matter to have to state that in 1913 50 men per 1,000 were admitted into hospital on account of syphilis or for gonorrhœa, or to admit that in France the admission rate for these diseases was 21 per 1,000, in Egypt 32 per 1,000, and at home 48 per 1,000. Those were awful figures, and at the present time deprived us of so much strength as to go a long way towards our losing the war. It was not nice to think of some seaports hesitating to adopt the treatment scheme, and the matter was so important that he thought public opinion should be brought to bear upon it. He warned the meeting that the statistics as to the incidence of these diseases in the Army did not really represent their prevalence, because a very large number of cases which ought to be classified under those heads were placed under others, though their course and their fatal termination were influenced by venereal contagion. These people went back to civil life, where, he believed, the diseases were more prevalent than in the Army, where facilities had long existed for treating them; and it was among the civilian population that they must be prevented.

### Mrs. Creighton said women must certainly know about these things, and she urged that they should try to know in the right way—not listen to panicmongers. The conditions were admittedly bad, but there were some people who, with little knowledge, would make them out to be worse than was actually the case. She also counselled women not to listen to those who suggested unwise ways of dealing with the question; great help would be obtained by a perusal of the literature issued by the Council. Knowledge should cause hope, not panic; and one of the things which had made her anxious with regard to the prominence given to this subject was just that feeling of panic which made people rush to suggest remedies which would result in making matters worse instead of better. There was a special work for women to do : a special work had been done by them in the past. They could never forget what one great woman—Josephine Butler—did to combat a system which was not only unjust to women, but one which had since been condemned by all enlightened persons as feeble and even disastrous in the results produced. The women of to-day had to carry on Josephine Butler's work, which meant to her almost martyrdom. The duty was pre-eminent because of the innocent victims of these diseases, the great majority women and children, and because it was by measures specially applied to women that ignorant, panic-struck people sought to remedy this evil. If—as, of course, was impossible—every diseased and infectious woman could be shut up that night, there would be on the next day a fresh number of infected and diseased women, because the men had not been shut up too. The diseases must be fought in two ways : first, by all the methods of cure which had been named; secondly, by prevention. Women had influence of various kinds in their own localities, and they could use it in favour of the establishment of treatment centres. Except in a few instances, infection was incurred wilfully—through folly, through sin, through ignorance—and London must care more about these matters. There should be more facilities for the sexes to meet under wholesome conditions, and much more must be done to secure for the people decent housing. Our young people must be taught to face and resist temptation, and not to rely upon either keeping temptation away or means of treatment. Before giving consent to marriage parents should carefully inquire whether the young man or woman had had venereal disease. Anxious inquiry was made as to worldly prospects and financial position, neither of which was so important. The education of midwives should also be extended, so that the number of cases of infantile blindness might be greatly lessened, and a strong public opinion should be created which would support all the efforts being made.

